# Avascular Necrosis of the Capitate

**Published:** 2017-06-05

**Authors:** David Buziashvili, Richard S Zeri, Tom Reisler

**Affiliations:** ^a^New Jersey Medical School, Rutgers University, Newark; ^b^Division of Plastic and Reconstructive Surgery, Department of Surgery, The Brody School of Medicine, East Carolina University, Greenville, NC

**Keywords:** avascular necrosis, osteonecrosis, capitate bone, Milliez classification, vascular supply carpus

## DESCRIPTION

A 41-year-old woman, right-hand-dominant hairdresser who was a smoker, presented with right wrist pain. Weeks prior to the magnetic resonance imaging (MRI) study, the patient was prescribed a course of oral prednisone for “arthritic pain.” No history of major trauma or inflammatory disease was reported.

## QUESTIONS

1. **What are the causes and predisposing factors to avascular necrosis (AVN) of the capitate?**
2. **What is the radiological classification of AVN of the capitate?**
3. **What are the signs and symptoms of this condition?**
4. **What are the nonsurgical and surgical treatments available for AVN of the capitate ?**

## DISCUSSION

Avascular necrosis of the capitate is extremely rare, with only 48 cases reported in the literature up to 2012.^1^ Trauma or repeated microtrauma is reported to be the cause in most cases. The remaining causes are idiopathic. In addition, other medical conditions have been associated with AVN of the capitate, including gout, Gaucher disease, and systemic lupus erythematosus. Local and systemic uses of corticosteroids have also been reported as a cause. Certain idiopathic capitate-AVN may be secondary to undocumented fractures of the capitate.^1^ The mean age of diagnosis is 27 years, with a range of 5 to 58 years. Men more often have a traumatic cause of capitate-AVN, whereas women are more often idiopathic,^1^ suggesting a possible autoimmune contribution. The vessels supplying the capitate enter distally and supply the capitate in a retrograde fashion both dorsally and volarly. In most cases, there is also a direct supply of its proximal pole through the volar capitate ligament. There are no significant vascular anastomoses in the capitate. There may be a possibility that there is an absence of the direct bloody supply in certain patients, predisposing them to capitate-AVN.

**Figure 1:**
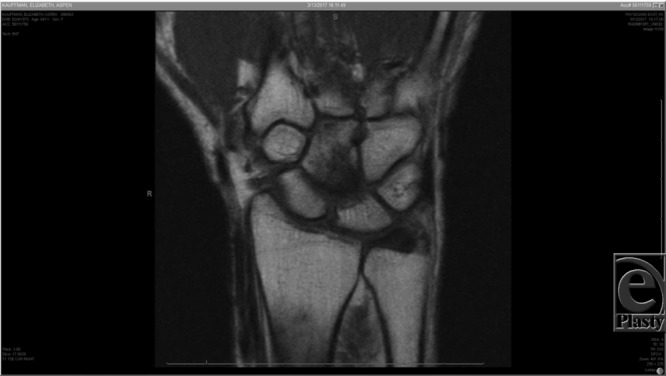
Coronal MRI slice of the right wrist. The proximal pole of the capitate has a low-intensity signal (Milliez type I).

**Figure 2:**
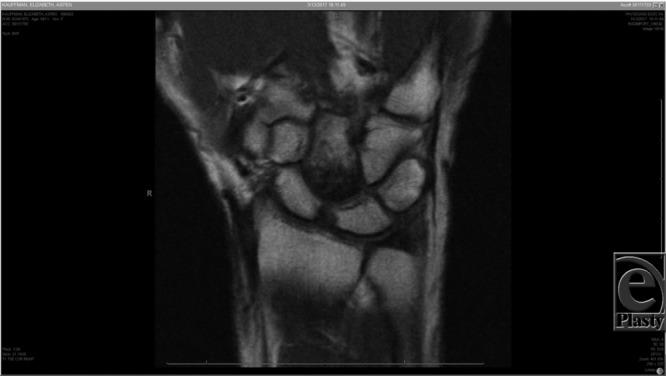
Coronal MRI slice of the right wrist. The proximal pole of the capitate has a low-intensity signal (Milliez type I).

**Figure 3:**
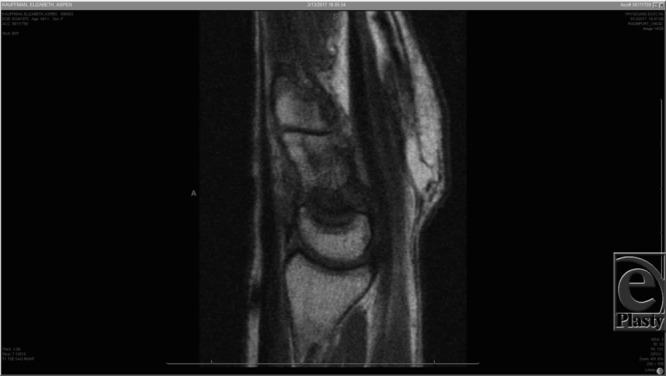
Sagittal MRI slice of the right wrist. The proximal pole of the capitate has a low-intensity signal (Milliez type I).

Capitate-AVN is typically diagnosed radiographically.^1^^-^3 Magnetic resonance imaging has been the preferred modality for diagnosis due to its high sensitivity (~95%), Figures 1–3, and it demonstrates changes before they are visible on a plain radiograph.^4^ Milliez et al^2^ proposed a radiographic classification system for this condition based on the location of involvement in the capitate (Table 1). It is important to note that the Milliez classification is descriptive and does not give guidance for clinical decision-making, nor prediction on outcome.

All reported cases of capitate-AVN presented with chronic dorsal wrist pain upon use or loading, reduced range of motion, stiffness, swelling, and crepitus.^1^

Treatment of capitate-AVN varies on the extent and location of necrosis.^1^ There is no consensus on treatment of capitate-AVN. The mainstay of the treatment is to reduce pain, as it is the most important factor for patient satisfaction,^1^ although range of motion and grip strength often remain unimproved.^1^^,^^5^ Although most cases are treated surgically, less invasive techniques have shown efficacy. Work modification and immobilization with a removable volar forearm cock-up splint have shown some success.^1^^,^^6^ Effective treatment of unfragmented capitate that has not collapsed or slightly collapsed includes vascularized bone grafting. There are several vascularized bone graft donor sites that have been utilized; these include distal radius bone graft based on the fourth extensor compartment artery with retrograde supply through the fifth extensor compartment artery,^1^ distal radius based on the 2,3 intercompartmental supraretinacular artery,^1^ vascularized medial femoral trochlea cartilage based on the descending geniculate artery,^5^ and vascularized bone graft from the iliac crest.^1^ Other options include resection of the affected area combined with bone grafting, or spacing with tendon or prosthesis.^1^^,^^3^ The mainstay of treatment of capitate-AVN in the presence of capitate and carpal collapse is carpal arthrodesis, usually with impaction of cancellous or corticocancellous bone graft from the iliac crest to optimize bone-healing potential.^1^ The goal is restoration of carpal height and position before fixation to minimize risk of adjoining degenerative disease.^1^^,^^3^ Some of the types of arthrodesis include scaphocapitolunate, capitolunate, capitohamate, 4-corner arthrodesis, or more elaborate midcarpal arthrodesis.^1^ Finally, total wrist arthrodesis, total wrist prosthesis, and denervation have been considered as salvage procedures.^1^

## Figures and Tables

**Table 1:** Milliez classification for radiological appearance of capitate-AVN*

Classification	Radiographic affected region
Type 1	Proximal pole
a	*Dome-shaped central lesion*
b	*Total head and neck*
c	*Radioproximal portion*
Type 2	Distal body
Type 3	Entire capitate

*AVN indicates avascular necrosis.
